# Lactate increases tumor malignancy by promoting tumor small extracellular vesicles production *via* the GPR81-cAMP-PKA-HIF-1α axis

**DOI:** 10.3389/fonc.2022.1036543

**Published:** 2022-12-01

**Authors:** Man Luo, Junqi Zhu, Jie Ren, Yuxiao Tong, Limin Wang, Shenglin Ma, Jiaoli Wang

**Affiliations:** ^1^ Department of Respiratory Medicine, Affiliated Hangzhou First People’s Hospital, Zhejiang University School of Medicine, Hangzhou, China; ^2^ The Fourth Clinical Medical College of Zhejiang Chinese Medical University, Hangzhou, China; ^3^ Department of Translation Medicine Center, Key Laboratory of Clinical Cancer Pharmacology and Toxicology Research of Zhejiang Province, Affiliated Hangzhou First People’s Hospital, Zhejiang University School of Medicine, Hangzhou, China; ^4^ Zhejiang University Cancer Centre, Hangzhou, China; ^5^ Department of Oncology, Affiliated Hangzhou Cancer Hospital, Zhejiang University School of Medicine, Hangzhou, China

**Keywords:** lactate, extracellular vesicles, tumor, GPR81, HIF-1α

## Abstract

Lactate and tumor cell-derived extracellular vesicles (TEVs) both contribute to tumor progression. However, it is still unclear whether lactate can accelerate tumor development by directly promoting TEV production. Here, we show that lactate decreases intracellular cAMP levels and subsequent PKA activation *via* GPR81, which inhibits the PKA-induced ubiquitination of HIF-1α that causes degradation. Then, the HIF-1α-mediated transcription of *Rab27a* is enhanced, leading to increased TEV release. In this way, lactate promotes lung metastasis by murine melanoma. In addition, we show that serum lactate levels are positively correlated with serum EV levels and Rab27a and HIF-1α protein levels in the tumor tissues of lung cancer patients. Thus, our results reveal a novel mechanism underlying lactate-mediated tumor progression induced by TEVs and provide new strategies for tumor therapy.

## Introduction

Unlike normal tissues, tumor tissues tend to generate ATP by the low-efficiency process of glycolysis even in the presence of enough oxygen, termed aerobic glycolysis, namely, the Warburg effect (1). Due to the Warburg effect, massive amounts of lactate accumulate in tumor tissues. Abnormal accumulation of lactate in tumor tissues contributes to poor tumor outcomes (2). Lactate is involved in promoting endothelial cell activation and angiogenesis (3, 4). Lactate induces an increased level of the oncogene c-Myc (5). Lactate also facilitates extracellular matrix remodeling and enhances cell migration by upregulating MMP-2 mRNA and protein expression (6). In addition, lactate is associated with tumor immunosuppression. Lactate has been reported to blunt T-cell responses to cancer (7). Lactate also increases VEGF and Arginase 1 expression in tumor-associated macrophages, polarizing these cells toward the immunosuppressive, tumor-promoting M2 phenotype (8). Recently, lactate was reported to mediate histone lactylation, resulting in M2-macrophage polarization (9). Therefore, inhibition of the production of lactate or prevention of the effects mediated by lactate is a promising therapeutic strategy for tumors, which suggests that further understanding of the diverse functions of this molecule in tumor progression is needed.

Extracellular vesicles (EVs) are roughly classified into ectosomes and exosomes. Ectosomes are vesicles generated by direct outward budding of the plasma membrane with diameters ranging from 50 nm to 1 μm. Exosomes, which have a relatively uniform size (30-150 nm in diameter), are small EVs (sEVs) released in an endosome-dependent manner (10). sEVs deliver different molecular cargos (lipids, proteins, DNA, mRNA, and microRNA) to recipient cells, thus affecting physiological or pathological processes, including tumor progression (11–13). Emerging evidence has revealed that tumors produce enhanced sEVs. sEV proteins are greatly increased in the plasma of melanoma patients (14). Compared with healthy controls, the plasma of prostate cancer patients contains more sEVs (15). Under tumor conditions, B cells secrete more CD19-positive sEVs (16). Although HIF-1α and Rab27a have been demonstrated to mediate increased sEV release (14, 16), the mechanisms responsible for enhanced sEV production are still largely unknown.

Microenvironmental acidity positively correlates with sEV release by tumor cells (15, 17, 18). *In vitro* experiments have shown that melanoma cells and prostate cancer cells cultured at pH 6.5 release more sEVs than the corresponding cells cultured at pH 7.4 (15, 18). The mechanism underlying the increased release of sEVs under acidic conditions has yet to be explored. Since lactate can create an acidic environment for tumors, we are interested in testing the possibility that lactate can directly induce sEV release by tumor cells.

Here, we demonstrated that lactate directly promoted sEV release from tumor cells *via* G-protein-coupled receptor 81 (GPR81). Lactate decreased the intracellular cAMP level and PKA activation in a GPR81-dependent manner. Subsequently, the PKA-mediated ubiquitination of HIF-1α leading to degradation was suppressed, resulting in the accumulation of HIF-1α proteins. Then, HIF-1α induced *Rab27a* transcription, thereby promoting sEV release. A positive correlation between lactate and sEV levels was observed in the plasma of cancer patients. Furthermore, the lactate levels in the plasma of cancer patients were positively correlated with the HIF-1α and Rab27a proteins in tumor tissues. Thus, our findings reveal that lactate, an essential metabolic product of tumors, can promote tumor development by increasing tumor sEV production.

## Results

### Lactate promotes sEV release from tumor cells

**Figure 1:**
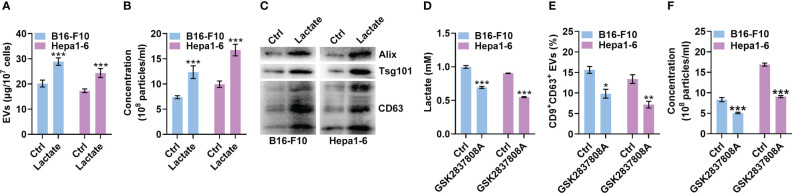
Lactate promotes sEV release from tumor cells. **(A-C)** A total of 1 × 10^7^ B16-F10 or Hepa1-6 cells were treated with 10 mM lactate for 24 h The sEVs in the culture supernatant were isolated. Then, the protein amount was measured by a BCA assay and statistically analyzed **(A)**, the particle concentration was measured by nanoparticle tracking analysis (NTA) **(B)**, and the sEV proteins were detected by western blotting **(C)**. **(D-F)** A total of 3 × 10^5^ B16-F10 or Hepa1-6 cells were treated with 1 μM GSK2837808A for 24 h The lactate level in the culture supernatants was measured **(D)**. Additionally, sEVs were captured with anti-CD63-coated latex beads, and the percentage of CD9^+^ sEVs was analyzed by flow cytometry **(E)**. The sEVs in the culture supernatant were isolated, and the particle concentration was measured by NTA **(F)**. Data are shown as the mean ± SD of one representative experiment (n = 3). Similar results were observed in three independent experiments. Unpaired Student’s *t* tests. *P < 0.05; **P < 0.01; ***P < 0.001.

The progression of melanoma and liver cancer has been demonstrated to be facilitated by the Warburg effect (19, 20). Therefore, we used mouse B16-F10 melanoma and Hepa1-6 liver cancer cells to evaluate the effect of lactate on sEV production by tumor cells. We cultured B16-F10 and Hepa1-6 cells in the presence of lactate *in vitro* and then semiquantitatively detected sEVs in the cell culture supernatants (21). Lactate promoted sEV release by B16-F10 and Hepa1-6 cells in a dose-dependent manner *in vitro* (**Figure S1A**), and treatment with 10 mM lactate did not affect the viability of either cell line (**Figure S1B**). A higher lactate concentration of 10 mM is reported in tumor tissues (22, 23), so 10 mM lactate was used in subsequent *in vitro* experiments. We further confirmed lactate promoted TEV production by assessing the total protein amount of sEVs, the particle concentration of sEVs and the levels of sEV proteins from equal numbers of cells (**Figures 1A-C**). In addition, lactate still increased the protein amounts of sEVs released from B16-F10 cells (B16-F10 EVs) after concentration with a sucrose gradient (**Figure S1C**). However, lactate showed no obvious effects on the morphology, protein content or size distribution of B16-F10 EVs (**Figures S1D-F**). To investigate the effect of autocrine lactate derived from tumor cells on sEV generation, we treated B16-F10 and Hepa1-6 cells with the lactic dehydrogenase (LDH) inhibitor GSK2837808A and found notably reduced lactate levels but no effect on viability for both cell lines (**Figures 1D**, **S1G**). Along with the reduction in lactate, CD9^+^CD63^+^ sEV production by B16-F10 and Hepa1-6 cells was significantly reduced (**Figure 1E**). Moreover, we confirmed that GSK2837808A also reduced the sEV numbers when assessed by NTA (**Figure 1F**). These results indicate that lactate positively regulates sEV generation by tumor cells.

### Lactate promotes sEV secretion by upregulating HIF-1α

**Figure 2:**
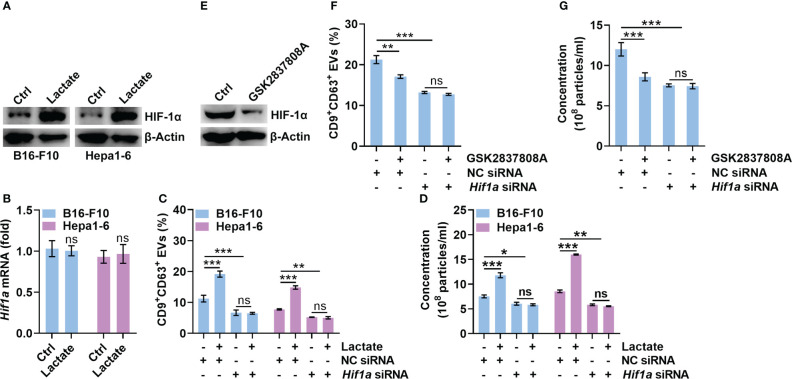
Lactate promotes sEV release in a HIF-1α-dependent manner. **(A, B)** B16-F10 and Hepa1-6 cells were treated with 10 mM lactate for 24 h The HIF-1α protein **(A)** and mRNA **(B)** levels were detected by western blotting **(A)** and real-time PCR. The *Hif1α* expression levels in Ctrl groups were set as 1, which was used as a baseline for other groups **(B)**. **(C, D)** After transfection with *Hif1a*-specific siRNA for 24 h, B16-F10 and Hepa1-6 cells were treated with 10 mM lactate for another 24 h The sEVs in the culture supernatant were captured with anti-CD63-coated latex beads, and the percentage of CD9^+^ sEVs was determined by flow cytometry and statistically analyzed **(C)**. The sEVs in the culture supernatant were isolated, and the particle concentration was measured by NTA **(D)**. **(E)** B16-F10 cells were treated with 1 μM GSK2837808A for 24 h The HIF-1α protein level was detected by western blotting. **(F, G)** After transfection with *Hif1a*-specific siRNA for 24 h, B16-F10 cells were treated with 1 μM GSK2837808A for another 24 h The sEVs in the culture supernatant were captured with anti-CD63-coated latex beads, and the percentage of CD9^+^ sEVs was determined by flow cytometry and statistically analyzed **(F)**. The sEVs in the culture supernatant were isolated, and the particle concentration was measured by NTA **(G)**. Data are shown as the mean ± SD of one representative experiment (n = 3). Similar results were observed in three independent experiments. One-way ANOVA followed by Tukey’s test. *P < 0.05; **P < 0.01; ***P < 0.001; ns, not significant.

HIF-1α has been reported to promote sEV release, while lactate can stabilize HIF-1α (8, 24). Therefore, we explored the role of HIF-1α in lactate-induced sEV release by tumor cells. We found that lactate increased the protein level of HIF-1α but not the mRNA level in B16-F10 and Hepa1-6 cells (**Figures 2A, B**). Then, we confirmed that HIF-1α silencing did not alter CD63 levels on B16-F10 EVs and sEVs from Hepa1-6 cells (Hepa1-6 EVs) (**Figures S2A, B**). However, HIF-1α silencing significantly reduced CD9^+^CD63^+^ sEVs or sEV numbers secreted by B16-F10 and Hepa1-6 cells (**Figures 2C, D**). In addition, lactate did not increase CD9^+^CD63^+^ sEVs or sEV numbers released from HIF-1α-silenced B16-F10 and Hepa1-6 cells (**Figures 2C, D**). In the presence of the HIF-1 inhibitor BAY87-2243, lactate no longer increased CD9^+^CD63^+^ sEVs or sEV numbers released from B16-F10 and Hepa1-6 cells (**Figures S2C, D**). In contrast, GSK2837808A treatment decreased HIF-1α protein levels in B16-F10 cells (**Figure 2E**). Consistent with this result, GSK2837808A treatment significantly inhibited CD9^+^CD63^+^ sEVs or sEV numbers released from B16-F10 cells (**Figures 2F, G**, **S2E, F**). Furthermore, in B16-F10 cells with HIF-1α silencing or BAY87-2243 treatment, GSK2837808A did not affect sEV secretion (**Figures 2F, G**, **S2E, F**). These data demonstrate that lactate increases sEV secretion in a HIF-1α-dependent fashion.

### Lactate upregulates HIF-1α *via* GPR81-mediated inhibition of the cAMP/PKA axis

**Figure 3:**
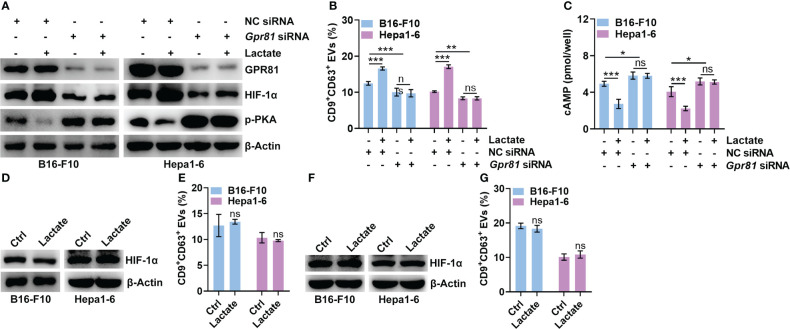
Lactate upregulates HIF-1α *via* GPR81-mediated inhibition of the cAMP/PKA axis. **(A-C)** After transfection with *Gpr81*-specific siRNA for 24 h, B16-F10 and Hepa1-6 cells were treated with or without 10 mM lactate for another 24 h Then, the protein levels of GPR81, HIF-1α, p-PKA and β-Actin in both cell lines were detected by western blotting **(A)**. The sEVs in the culture supernatant were captured with anti-CD63-coated latex beads, and the percentage of CD9^+^ sEVs was determined by flow cytometry and statistically analyzed **(B)**. The intracellular cAMP concentration was measured **(C)**. **(D, E)** In the presence of 1 mM SQ22536, B16-F10 and Hepa1-6 cells were treated with or without 10 mM lactate for 24 h Then, HIF-1α and β-Actin in both cell lines were detected by western blotting **(D)**. The sEVs in the culture supernatant were captured with anti-CD63-coated latex beads, and the percentage of CD9^+^ sEVs was determined by flow cytometry and statistically analyzed **(E)**. **(F, G)** In the presence of 10 μM H-89, B16-F10 and Hepa1-6 cells were treated with or without 10 mM lactate for 24 h Then, HIF-1α and β-Actin in both cell lines were detected by western blotting **(F)**. The sEVs in the culture supernatant were captured with anti-CD63-coated latex beads, and the percentage of CD9^+^ sEVs was determined by flow cytometry and statistically analyzed **(G)**. Data are shown as the mean ± SD of one representative experiment (n = 3). Similar results were observed in three independent experiments. One-way ANOVA followed by Tukey’s test in b, c; unpaired Student’s *t* tests in e, **(G)** *P < 0.05; **P < 0.01; ***P < 0.001; ns, not significant.

GPR81 and GPR132 have been reported to be lactate receptors (25, 26). Next, we determined the roles of GPR81 and GPR132 in the lactate-mediated upregulation of HIF-1α. Knockdown of GPR81 reduced HIF-1α and CD9^+^CD63^+^ sEV release from B16-F10 and Hepa1-6 cells (**Figures 3A, B**). However, the knockdown of GPR132 did not cause these effects (**Figures S3A, B**). In addition, the knockdown of GPR81 but not GPR132 abolished the lactate-induced increases in HIF-1α and CD9^+^CD63^+^ sEV production in B16-F10 and Hepa1-6 cells (**Figures 3A, B**, **S3A, B**). Activation of GPR81 by lactate decreases intracellular cAMP levels and inhibits PKA activity (27). We also found that the knockdown of GPR81 but not GPR132 enhanced PKA activation and cAMP levels in B16-F10 and Hepa1-6 cells (**Figures 3A, C**, **S3A, C**). Consistently, the knockdown of GPR81 but not GPR132 eliminated the lactate-mediated suppression of PKA activation and decreased the cAMP level (**Figures 3A, C**, **S3A, C**). To determine the effect of the cAMP/PKA axis on lactate-induced HIF-1α upregulation, we treated B16-F10 and Hepa1-6 cells with the cAMP inhibitor SQ22536 and found notably increased HIF-1α protein levels in the treated B16-F10 and Hepa1-6 cells (**Figure S3D**). Accordingly, SQ22536 promoted sEV release from B16-F10 and Hepa1-6 cells (**Figure S3E**). In the presence of SQ22536, lactate no longer upregulated HIF-1α or promoted CD9^+^CD63^+^ sEV release in either cell line (**Figures 3D, E**). The PKA inhibitor H-89 also obviously increased the HIF-1α protein levels and CD9^+^CD63^+^ sEV production of B16-F10 and Hepa1-6 cells (**Figures S3F, G**). In addition, H-89 also abrogated the lactate-induced increases in HIF-1α and CD9^+^CD63^+^ sEV production in both cell lines (**Figures 3F, G**). These results suggest that lactate increases HIF-1α protein levels in tumor cells *via* the GPR81/cAMP/PKA axis.

### Lactate inhibits the PKA-mediated ubiquitination degradation of HIF-1α

**Figure 4:**
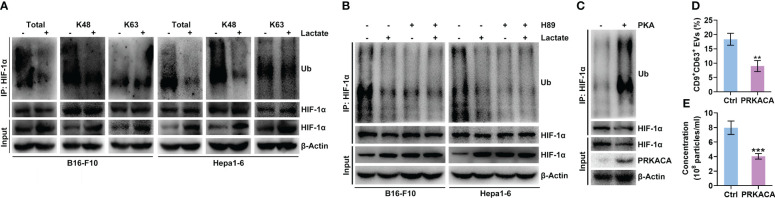
Lactate inhibits the PKA-mediated ubiquitination of HIF-1α that causes degradation. **(A)** After treatment with or without 10 mM lactate for 24 h, the total, K48-linked and K63-linked polyubiquitination of HIF-1α in B16-F10 and Hepa1-6 cells was detected by western blotting. **(B)** In the presence of 10 μM H-89, B16-F10 and Hepa1-6 cells were treated with or without 10 mM lactate for 24 h Then, the K48-linked polyubiquitination of HIF-1α in both cell lines was detected by western blotting. **(C-E)** Expression vectors for Ub-HA and HIF-1α-Flag were transfected into HEK293 cells along with PRKACA-expressing or empty control vectors for 24 h Then, K48-linked polyubiquitination was detected by western blotting **(C)**. The sEVs in the culture supernatant were captured with anti-CD63-coated latex beads, and the percentage of CD9^+^ sEVs was determined by flow cytometry and statistically analyzed **(D)**. The sEVs in the culture supernatant were isolated, and the particle concentration was measured by NTA **(E)**. Data are shown as the mean ± SD of one representative experiment (n = 3). Similar results were observed in three independent experiments. Unpaired Student’s *t* tests. **P < 0.01; ***P < 0.001.

Lactate did not increase the *Hif1a* mRNA level in B16-F10 or Hepa1-6 cells (**Figure 2B**). Furthermore, lactate did not affect the HIF-1α protein level in B16-F10 or Hepa1-6 cells treated with proteasome inhibitor MG132 (**Figure S4A**), indicating the posttranslational regulation of HIF-1α by lactate. Ubiquitin-mediated proteolysis of HIF-1α is well defined (28). We found that lactate reduced the total and K48-linked polyubiquitination of HIF-1α but did not change the K63-linked polyubiquitination in B16-F10 and Hepa1-6 cells (**Figure 4A**). K48 chains tag substrates for proteasomal degradation, whereas K63 chains act as proteasome-independent signals (29). These results suggest that lactate inhibits the ubiquitin-mediated proteolysis of HIF-1α. PKA has been reported to participate in the regulation of protein ubiquitination (30, 31). H-89 treatment did not increase the *Hif1a* mRNA level in B16-F10 or Hepa1-6 cells (**Figure S4B**). Therefore, we assumed that PKA promoted the ubiquitination-mediated degradation of HIF-1α induced, which was attenuated by lactate. As expected, the H-89 treatment markedly decreased the K48-linked polyubiquitination of HIF-1α in B16-F10 and Hepa1-6 cells (**Figure S4C**). In addition, the H-89 treatment completely abolished the lactate-induced decrease in the K48-linked polyubiquitination of HIF-1α in both cell lines (**Figure 4B**). To further confirm the role of PKA in HIF-1α ubiquitination, HEK293 cells were transfected with PKA catalytic subunit α (PRKACA)-expressing vectors, and the polyubiquitination of HIF-1α was obviously enhanced while the HIF-1α protein level was decreased in cells with PRKACA overexpression (**Figure 4C**). In line with these results, we found a significant decrease in sEV release from HEK293 cells with PRKACA overexpression (**Figures 4D, E**). Altogether, these results indicate that lactate suppresses the ubiquitin-mediated proteolysis of HIF-1α by inactivating PKA.

### Lactate promotes tumor progression by inducing sEV release from tumor cells

**Figure 5:**
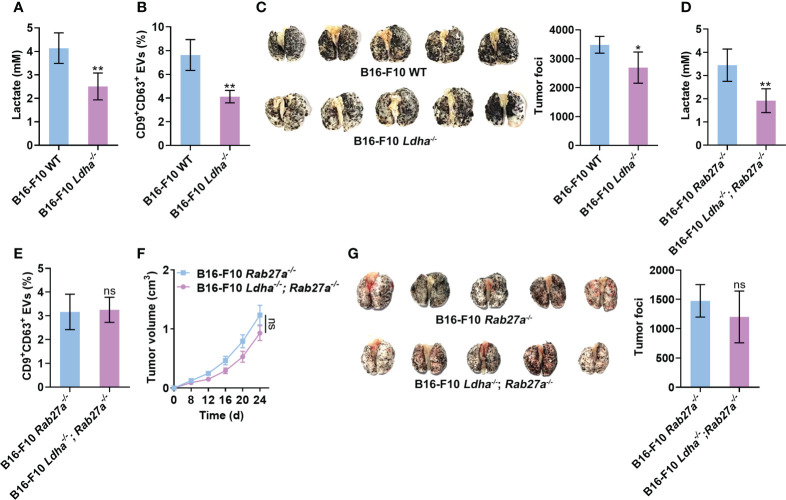
Lactate promotes tumor progression by inducing sEV release from tumor cells. **(A-C)** Mice received a subcutaneous injection of B16-F10 WT or B16-F10 *Ldha^-/-^* cells on Day 0. Then, the lactate levels in the plasma of these mice were measured **(A)**. Additionally, the serum sEVs of these mice were captured with anti-CD63-coated latex beads, and the percentage of CD9^+^ sEVs was determined by flow cytometry and statistically analyzed **(B)**. Lung metastasis was macroscopically (left) or statistically analyzed (right) on Day 35 **(C)**. **(D-G)** Mice received a subcutaneous injection of B16-F10 *Rab27a^-/-^* cells or B16-F10 *Ldha^-/^*; *Rab27a^-/-^* cells on Day 0. Then, the lactate levels in the plasma of these mice were measured **(D)**. Additionally, the serum sEVs were captured with anti-CD63-coated latex beads, and the percentage of CD9^+^ sEVs was determined by flow cytometry and statistically analyzed **(E)**. Tumor size was measured **(F)**, and lung metastasis was macroscopically (left) or statistically analyzed (right) on Day 35 **(G)**. Data are shown as the mean ± SD of one representative experiment (n = 5). Similar results were observed in three independent experiments. Unpaired Student’s *t* tests. *P < 0.05; **P < 0.01; ns, not significant.

Next, we wanted to substantiate the effects of lactate on tumor cell sEV release and tumor progression *in vivo*. To constantly inhibit lactate generation in B16-F10 cells, we constructed B16-F10 cells with LDHA knocked out (B16-F10 *Ldha^-/-^* cells) because LDHA is the predominantly enzymatic form of LDH in cancer cells (32). We confirmed that B16-F10 *Ldha^-/-^* cells secreted fewer CD9^+^CD63^+^ sEVs than B16-F10 cells without LDHA knocked out (B16-F10 WT cells) *in vitro* (**Figures S5A, B**). Consistent with a previous publication (33), B16-F10 *Ldha^-/-^* cells showed no noticeable difference in proliferation *in vitro* but produced primary tumors exhibiting slower growth than primary tumors established with B16-F10 WT cells *in vivo* (**Figures S5C, D**). The levels of lactate and CD9^+^CD63^+^ sEVs in the plasma of mice transplanted with B16-F10 *Ldha^-/-^* cells were reduced (**Figures 5A, B**). In addition, a decrease in tumor lung metastasis was observed in mice transplanted with B16-F10 *Ldha^-/-^* cells (**Figure 5C**). To evaluate the role of sEVs in this process, we constructed Rab27a-knockout (B16-F10 *Rab27a^-/-^* cells) and LDHA and Rab27a double-knockout (B16-F10 *Ldha^-/^*; *Rab27a^-/-^* cells) B16-F10 cells and found that the CD9^+^CD63^+^ sEV levels produced by B16-F10 *Ldha^-/^*; *Rab27a^-/-^* cells were comparable to those produced by B16-F10 *Rab27a^-/-^* cells *in vitro* (**Figures S5A, E**). A higher lactate level could be detected in the plasma of mice transplanted with B16-F10 *Rab27a^-/-^* cells than in that of mice transplanted with B16-F10 *Ldha^-/^*; *Rab27a^-/-^* cells (**Figure 5D**). However, the CD9^+^CD63^+^ sEVs in the plasma showed no significant difference between the two groups of mice (**Figure 5E**). Although the growth of B16-F10 *Ldha^-/^*; *Rab27a^-/-^* tumors was slightly slower than that of B16-F10 *Rab27a^-/-^* tumors, the difference was insignificant (**Figure 5F**). In addition, similar results for tumor lung metastasis were obtained for the two groups of mice (**Figure 5G**). Together, these results suggest that sEVs probably involve lactate-induced tumor progression.

### Plasma lactate and sEVs of cancer patients are positively correlated

**Figure 6:**
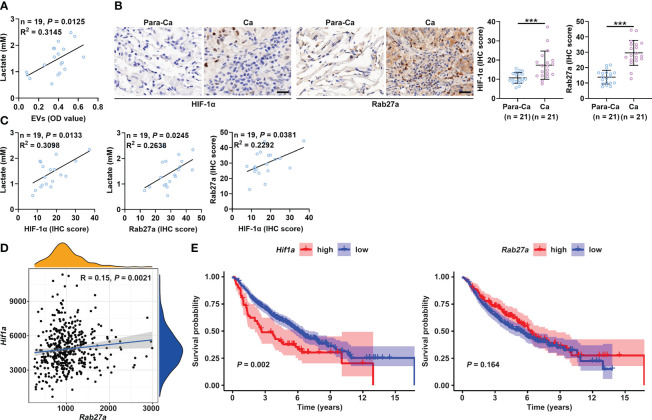
Plasma lactate and sEVs of cancer patients are positively correlated. **(A)** Correlation analysis of the levels of lactate and sEVs in the plasma of lung cancer patients. **(B)** The HIF-1α and Rab27a protein levels in Ca and Para-Ca of lung cancer patients were detected by IHC staining (left) and statistically analyzed. Scale bar, 20 μm. **(C)** Correlation analysis of the indicated factors. **(D)** Correlation of *Hif1a* and *Rab27a* mRNA levels. **(E)** Prognostic analysis of lung cancer patients with high or low *Hif1a* and *Rab27a* mRNA levels. Data are shown as the mean ± SD. Spearman correlation analysis in **(A, C, D)**; unpaired Student’s *t* tests in **(B)**; and log-rank test in **(E)**. ***P < 0.001.

Compared to healthy controls, cancer patients have been reported to have increased lactate and sEV levels in the plasma (14, 34). Therefore, we analyzed the relationship between the plasma levels of lactate and sEVs in cancer patients to verify our findings. First, we confirmed that the plasma levels of lactate and sEVs were significantly higher in lung cancer patients than in healthy controls (**Figures S6A, B** and **Supplementary Table 1**). A significant positive correlation was observed between the levels of lactate and sEVs in the plasma of lung cancer patients (**Figure 6A**). Immunohistochemical (IHC) staining results showed that the HIF-1α protein levels in cancerous tissues (Ca) from lung cancer patients were higher than those in paracancerous tissues (Para-Ca) (**Figure 6B**). HIF-1α is reported to increase sEV secretion by B cells of tumor mice in a manner dependent on Rab27a (16). Consistent with this report, we found that HIF-1α overexpression significantly increased the expression of Rab27a mRNA and protein levels in B16-F10 and Hepa1-6 cells (**Figures S6C, D**). We also found higher Rab27a protein levels in the Ca of lung cancer patients (**Figure 6B**). Plasma lactate levels and the HIF-1α and Rab27a protein levels in Ca were positively correlated in a pairwise analysis (**Figure 6C**). Although the protein levels of smpd3, another regulator of sEV production (35), were also significantly increased in Ca compared with Para-Ca (**Figure S6E**), plasma lactate levels and HIF-1α were not correlated with smpd3 (**Figure S6F**). Furthermore, results from the TCGA database suggested that *Hif1a* and *Rab27a* mRNA levels were also positively correlated (**Figure 6D**). In addition, higher mRNA expression of *Hif1a* was associated with poorer survival in lung cancer patients (**Figure 6E**). However, the mRNA expression levels of *Rab27a* did not seem to affect the survival of lung cancer patients (**Figure 6E**). Altogether, these data indicate the important role of lactate-induced sEVs in the progression of cancer patients.

## Discussion

Accumulating evidence has demonstrated that lactate in the tumor microenvironment is not an innocuous bystander metabolite but has special roles in tumor progression (36). Lactate not only promotes tumor growth by inducing tumor angiogenesis but also suppresses antitumor immune responses (3, 4, 7, 37). TEVs are critical in tumor growth and metastasis (38). In this study, we demonstrated that lactate could directly promote tumor cell release of sEVs after activating GPR81, leading to increased malignancy. This finding represents a novel mechanism by which lactate promotes tumor progression. It also supports the idea that “aerobic glycolysis”, a pathway that generates ATP with low efficiency, is selected by tumor evolution. In this way, tumors produce sufficient lactate to meet aggressive growth demands. Our results further underline that lactate is an important target for preventing tumor progression as a cancer cell-specific metabolite.

Compared with mice bearing B16-F10 WT tumors, decreased levels of lactate and sEVs could be observed in mice bearing B16-F10 *Ldha^-/-^* tumors. Accordingly, the growth and metastasis of B16-F10 *Ldha^-/-^* tumors were substantially inhibited. Compared with B16-F10 *Rab27a^-/-^* tumor-bearing mice, B16-F10 *Ldha^-/^*; *Rab27a^-/-^* tumor-bearing mice showed a reduction in only the lactate level, not the sEV level. Significant differences in tumor growth and metastasis were not observed between these two groups of mice. The lactate-mediated promotive effect on tumor progression depended entirely on sEVs. However, the mechanisms underlying lactate-induced tumor progression are complicated. Similar to lactate, sEVs induce tumor angiogenesis and suppress antitumor immune responses (39). These effects of lactate may also be sEV dependent. Nevertheless, it is noteworthy that the growth and metastasis of B16-F10 *Ldha^-/-^*; *Rab27a^-/-^* tumors were indeed slightly inhibited compared with those of B16-F10 *Ldha^-/-^* tumors, although the differences were not significant. Thus, increased sEV release from tumor cells may not be the unique factor accounting for lactate-mediated tumor progression.

The metabolic switch pertinent to accelerated glycolysis in cancer cells results from increased expression of oncogenes, primarily c-Myc and HIF-1α (40). The conversion of pyruvic acid to lactic acid catalyzed by LDHA is required to sustain the increased rates of glycolysis in cancer cells. LDHA is almost universally upregulated in cancer cells by c-Myc and HIF-1α (36). Lactate has also been reported to induce HIF-1α accumulation (8). Therefore, lactate probably exerts a positive feedback effect in HIF-1α upregulation. Our results indicate that lactate promotes sEV release from tumor cells in a HIF-1α-dependent manner. Thus, the positive feedback effect of lactate involved in HIF-1α upregulation will further accelerate tumor cell release of sEVs and thus increase tumor aggressiveness. Hence, breaking the positive feedback loop between lactate and HIF-1α represents a valuable “key” to preventing sEV production in tumor cells, thus benefiting tumor therapy.

The phosphorylation of E3 ubiquitin ligases is critical to their functions. The ERK-mediated phosphorylation of the E3 ubiquitin ligases MARCH5, MULAN, and Parkin results in their activation (41). The RSK2-mediated phosphorylation of TRAF6 is required for its K63 ubiquitination (42). PKA is also reported to inactivate the Cul4-DDB1 E3 ubiquitin ligase complex by phosphorylating DDB1 (43). We found that PKA promoted HIF-1α ubiquitination and its subsequent proteolysis. Although we did not identify the E3 ubiquitin ligase responsible for HIF-1α ubiquitination in this process, it can be deduced that PKA probably phosphorylates a certain E3 ubiquitin ligase and activates it, causing increased HIF-1α ubiquitination. PKA can directly phosphorylate NEK10, an E3 ubiquitin ligase substrate, leading to NEK10 degradation (44). Alternatively, PKA may affect HIF-1α ubiquitination by directly phosphorylating HIF-1α.

In summary, we reveal the critical role of lactate in sEV release from tumor cells and elucidate the corresponding mechanism in this study. Our findings emphasize that tumors can utilize their specific metabolic characteristics to increase sEV generation and thereby promote tumor growth. Thus, blocking lactate-induced sEV release will be an ideal strategy for tumor therapy.

## Materials and methods

### Reagents

A lactate detection kit (MAK064) was purchased from Sigma–Aldrich (St. Louis, MO, USA). A cAMP detection kit (KGE012B) was purchased from R&D Systems (Minneapolis, MN, USA). GSK2837808A (HY-100681), BAY87-2243 (HY-15836), SQ22536 (HY-100396), H-89 (HY15979) and MG132 (HY-13259) were obtained from MedChemExpress (Monmouth Junction, NJ, USA). Anti-CD9 antibody (MZ3) and CD63 antibody (NVG-2) were purchased from BioLegend (San Diego, CA, USA). HIF-1α-Flag (MR210895) and PRKACA (MR205322) vectors were obtained from Origene Technologies (Rockwell, Maryland, USA).

### Human samples

Human lung tissue samples from lung cancer patients and Para-Ca were obtained from the Affiliated Hangzhou First People’s Hospital, Zhejiang University School of Medicine. The Affiliated Hangzhou First People’s Hospital Ethics Committee approved the collection of human samples and patient information. All the patients were informed of the use of their samples, and signed consent forms were obtained.

### Mice and cell lines

Female C57BL/6J mice (6-8 weeks old) were purchased from Joint Ventures Sipper BK Experimental Animal Co. (Shanghai, China). Mice were housed in a specific pathogen-free facility, and the experimental protocols were approved by the Animal Care and Use Committee of the School of Medicine, Zhejiang University. Murine B16-F10 cells, Hepa1-6 cells and HEK293 cells were obtained from American Type Culture Collection (Manassas, VA, USA). The cells were cultured in Dulbecco’s modified Eagle’s medium (DMEM) supplemented with 10% (v/v) fetal bovine serum (FBS) and 100 μg/ml penicillin-streptomycin. All cells were maintained at 37°C in a humidified atmosphere containing 5% CO_2_.

### sEV isolation

Conditioned media were collected from B16-F10 cells and Hepa1-6 cells at approximately 90% confluence in 10-cm cell culture dishes; the cells were cultured in DMEM containing FBS depleted of bovine serum sEVs. The collected media were then differentially centrifuged at 300 × *g* for 10 min, 1,200 × *g* for 20 min, and 10,000 × *g* for 30 min at 4°C. The supernatants from the final centrifugation were ultracentrifuged at 100,000 × *g* and 4°C for 1 h. After removing the supernatants, the exosomal pellets were washed with a large volume of ice-cold PBS and centrifuged at 100,000 × *g* and 4°C for 1 h. The final pellets were resuspended in ice-cold PBS. The concentrations of exosomal proteins were measured using a BCA Protein Assay Kit (23225, Thermo Fisher Scientific; Waltham, CA, USA) according to the manufacturer’s instructions.

### NTA

To measure particle size and concentration, sEVs were analyzed by NTA using a NanoSight NS300 system configured with a 488-nm laser and high-sensitivity sCMOS camera, with the results analyzed with NTA 3.2 software (Malvern PANalytical, Malvern, UK).

### Flow cytometry

To detect sEVs in cell culture supernatants, supernatants containing FBS depleted of bovine serum sEVs were cleared by centrifugation at 300 × *g* for 10 min and 2,000 × *g* for 20 min. Four-micrometer aldehyde sulfate beads were first coated with purified anti-CD63 antibodies and then blocked with FBS depleted of bovine serum sEVs at room temperature (RT) for 1 h. The beads were washed twice in PBS and centrifuged at 3000 × *g* for 5 min. The cleared supernatants (1 ml) were incubated with anti-CD63-coupled beads overnight at 4°C with shaking. The beads were washed twice in PBS and incubated with anti-CD9 antibodies for 30 min at 4°C. After washing twice in PBS, the beads were acquired on an ACEA NovoCyte, and the data were analyzed with NovoExpress software (ACEA Biosciences, San Diego, CA, USA).

### Electron microscopy

sEVs were diluted in 100 μl of PBS, and 20 μl of suspension was placed onto formvar carbon-coated copper grids at RT for 1 min. Any excess suspension was removed using filter paper. The sEVs were stained with 2% phosphotungstic acid at RT for 5 min. The grids were then fixed with 2.5% glutaraldehyde at RT for 5 min, followed by rinsing with PBS 3 times. Images were observed with a Philips Tecnai-10 transmission electron microscope (Hanover, MD, USA) operating at 80 kV.

### Detection of lactate levels

The supernatants from B16-F10 or Hepa1-6 cells with different treatments, and the plasma from tumor mice or cancer patients were collected. Then, the lactate levels were measured with a lactate detection kit from Sigma–Aldrich according to the manufacturer’s instructions.

### Detection of cAMP levels

The supernatants from B16-F10 or Hepa1-6 cells with different treatments were collected, and the cAMP levels were measured with a cAMP detection kit from R&D Systems according to the manufacturer’s instructions.

### Immunoblot analysis and immunoprecipitation

A total of 10 μg of sEVs or crude proteins extracted from cell lysates was separated by 10% SDS–PAGE and transferred to a polyvinylidene difluoride membrane (Millipore). The membrane was blocked with 5% BSA in TBST and then incubated with appropriate primary antibodies overnight at 4°C. After incubating with HRP-coupled secondary antibodies for 1 h, the membrane was scanned using a Tanon 4500 (Shanghai, China). For complex coimmunoprecipitation, cell extracts were prepared by using lysis buffer (50 mM Tris, pH 7.4; 150 mM NaCl; 0.5% (vol/vol) Nonidet P-40; 1 mM EDTA) supplemented with a protease inhibitor cocktail (Roche). The lysates were incubated with the anti-flag (M2)-agarose or antibody-coupled beads for 4 h at 4°C. The immunoprecipitates were washed three times with the same buffer and subjected to immunoblot analysis. The antibodies information were listed below: anti-GRP94 (EPR22847-50, 1:3000), anti-Tsg101 (EPR7130(B), 1:3000), anti-Alix (EPR15314, 1:3000), anti-CD63 (EPR21151, 1:3000), anti-Rab27a (ab55667, 1:3000), anti-HIF-1α (EPR16897, 1:3000), anti-p-PKA (EP2606Y, 1:3000), anti-PRKACA (EP2102Y, 1:3000), anti-UB (EPR8830, 1:3000), anti-K63-UB (EPR8590-448, 1:3000), anti-K48-UB (EP8589, 1:3000), anti-LDHA (EP1566Y, 1:3000), anti-GAPDH (ab8245, 1:3000) and anti-β-Actin (ab8226, 1:3000) antibodies were purchased from Abcam (Cambridge, MA, USA). Anti-GPR81 (20146-1-AP, 1:1000) and anti-GPR132 (17026-1-AP, 1:1000) were from Proteintech (Wuhai, Hubei, China).

### Plasmid and siRNA transfection

Cells were transfected with plasmids using JetPEI^®^ Transfection Reagent (Polyplus Transfection) according to the manufacturer’s protocol. The transient transfection of siRNA into cells was performed with INTERFERin^®^ Transfection Reagent (Polyplus Transfection) according to the manufacturer’s instructions. The siRNAs including sequences targeting murine *Hif1a* (sc-35562), *Gpr132* (sc-44371), and *Gpr81* (sc-44644) and NC siRNA (sc-37007) were obtained from Santa Cruz Biotechnology (Santa Cruz, CA, USA).

### Real-time PCR

Total RNA was extracted using TRIzol reagent (9109, TaKaRa, Kusatsu, Shiga, Japan) and reverse transcribed into cDNA using a cDNA synthesis kit (RR047A, TaKaRa) according to the manufacturer’s instructions. Real-time PCR was conducted using SYBR Green (RR420, TaKaRa). The following thermal cycling conditions were used for PCR: 1 cycle at 95°C for 30 s followed by 40 cycles at 95°C for 5 s and 60°C for 34 s. *Actb* was used as the internal control. The relative mRNA expression of indicated genes were calculated using the 2^−ΔΔCT^ method with the normalization to *Actb* expression.

### Stable cell line construction

For depletion of LDHA or Rab27a in B16-F10 cells, a guide RNA plasmid (encoding an LDHA sgRNA or a Rab27a sgRNA) and Cas9-IRES-EGFP plasmid (encoding Cas9 and GFP) (Sigma–Aldrich) were cotransfected into B16-F10 cells. After cotransfection of the two plasmids into B16-F10 cells, GFP-positive cells were sorted using a Beckman Coulter DxFLEX flow cytometer (Beckman Coulter, Inc.). After sorting, single cells were cultured in 96-well plates. The LDHA- or Rab27a-knockout efficiency was confirmed by IB analysis. For depletion of both LDHA and Rab27a in B16-F10 cells, the two guide RNA plasmids and Cas9-IRES-EGFP plasmid were cotransfected into B16-F10 cells.

### Tumor models and treatments

Mice were subcutaneously injected with 1 × 10^6^ B16-F10, B16-F10 *Ldha^-/-^*, B16-F10 *Rab27a^-/-^*, or B16-F10 *Ldha^-/^*; *Rab27a^-/-^* cells on Day 0. Mouse plasma was collected to detect the serum levels of lactate and sEVs on Day 10. The mice were euthanized, and lung metastasis was macroscopically or statistically analyzed on Day 35. Tumor growth was monitored every four days by measuring tumor length and width. Tumor volume was calculated according to the following equation: length × width × 0.5 × width.

### IHC staining

Lung tissues dissected from lung cancer patients were subjected to IHC staining by Servicebio (Wuhan, Hubei, China). Images of the sections were captured, and positive areas were analyzed. The intensity and extent of HIF-1α, Rab27a and Smpd3 were quantified by ImageJ analysis with the IHC profiler plugin (NIH, Bethesda, MD, USA). Cytoplasmic expression were quantified using a four-value intensity score (0, none; 1+, weak; 2+, moderate; and 3+, strong) and the percentage (0-100%) of the extent of reactivity. A final expression score was obtained by multiplying the intensity and reactivity extension values (range, 0-300).

### Cell proliferation assay

B16-F10 cells or Hepa1-6 cells were treated with 10 mM lactate for 24 h. Then, 10 μl of CCK8 reagent was added to each well, and the cells were incubated for an additional 2 h. The absorbance of each well was read at 450 nm.

### Statistical analysis

All statistical analyses were performed using Prism 8.0 software (San Diego, CA, USA). Results are expressed as the mean ± s.d. Unpaired Student’s *t* test was used to compare differences between two groups. One-way ANOVA followed by the Tukey’s test was used to compare differences among multiple groups. The log-rank test was used for survival analysis, and the Spearman rank-order correlation test was used for Pearson correlation analysis. Differences were considered statistically significant at *P* < 0.05.

## Data availability statement

The raw data supporting the conclusions of this article will be made available by the authors, without undue reservation.

## Ethics statement

The studies involving human participants were reviewed and approved by Affiliated Hangzhou First People’s Hospital ethics committee. The patients/participants provided their written informed consent to participate in this study. The animal study was reviewed and approved by Animal Care and Use Committee of the School of Medicine, Zhejiang University. Written informed consent was obtained from the individual(s) for the publication of any potentially identifiable images or data included in this article.

## Author contributions

ML, JZ, JR, YT, and LW performed various experiments. SM and JW designed the project and supervised the study. JZ and JW wrote the manuscript. All authors contributed to the article and approved the submitted version.

## Funding

This work was supported by the Major Project of Hangzhou Health Science and Technology Plan (Z20200134) and Joint Preresearch Fund for Clinical Scientific Research of Hangzhou First People’s Hospital Affiliated with Zhejiang University (YYJJ2019Z07).

## Conflict of interest

The authors declare that the research was conducted in the absence of any commercial or financial relationships that could be construed as a potential conflict of interest.

## Publisher’s note

All claims expressed in this article are solely those of the authors and do not necessarily represent those of their affiliated organizations, or those of the publisher, the editors and the reviewers. Any product that may be evaluated in this article, or claim that may be made by its manufacturer, is not guaranteed or endorsed by the publisher.
