# Supplementary material for: Lactate increases tumor malignancy by promoting tumor small extracellular vesicles production via the GPR81-cAMP-PKA-HIF-1α axis
Source: Front Oncol. 2022 Dec 1;12:1036543. doi: 10.3389/fonc.2022.1036543 (PMC9753130; doi:10.3389/fonc.2022.1036543)
Supplement: Supplementary file 1 [file DataSheet_1.docx]

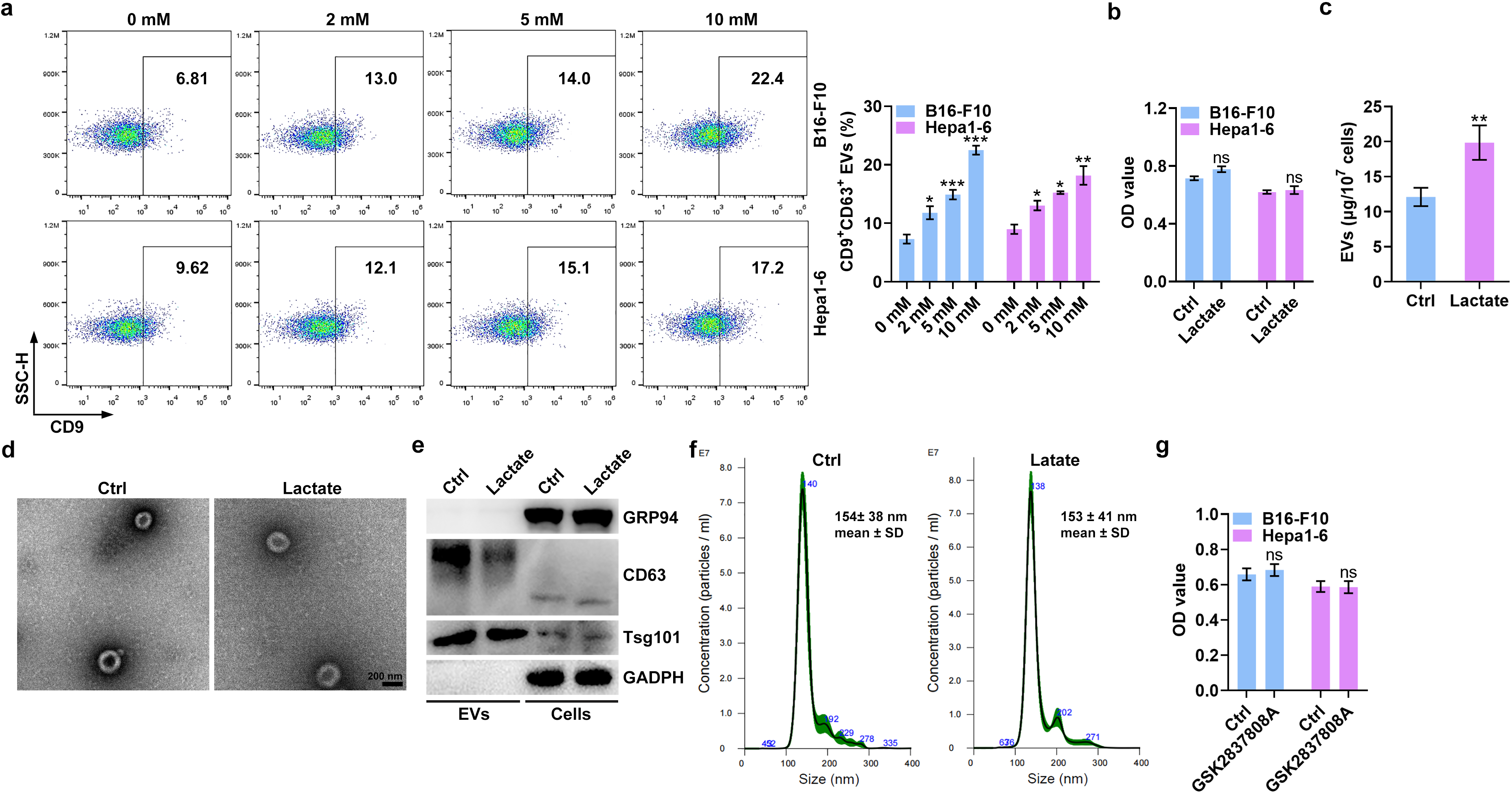


**Figure S1.** ***Lactate promotes sEV release from tumor cells.*** (a) B16-F10 or Hepa1-6 cells (3 × 10^5^) were treated with 2, 5 and 10 mM lactate for 24 h. The sEVs were captured with anti-CD63-coated latex beads, and the percentage of CD9^+^ sEVs was determined by flow cytometry (left) and statistically analyzed (right). Latex beads were gated based on isotype control. (b) B16-F10 or Hepa1-6 cells (3 × 10^5^) were treated with 10 mM lactate for 24 h, and cell viability was determined with a CCK-8 assay. (c) The protein amounts of B16-F10 cell-derived sEVs after sucrose gradient concentration were quantified with a BCA assay. (d-f) The morphology (d), protein content (e) and size distribution (f) of the same amount of sEVs from B16-F10 cells were detected by electron microscopy (d), western blotting (e) and NTA (f), respectively. (g) B16-F10 or Hepa1-6 cells (3 × 10^5^) were treated with 1 μM GSK2837808A for 24 h, and cell viability was determined with a CCK-8 assay. Data are shown as the mean ± SD of one representative experiment (n = 3). Similar results were observed in three independent experiments. Unpaired Student’s t tests. **P* < 0.05; ***P* < 0.01; ****P* < 0.001; ns, not significant.


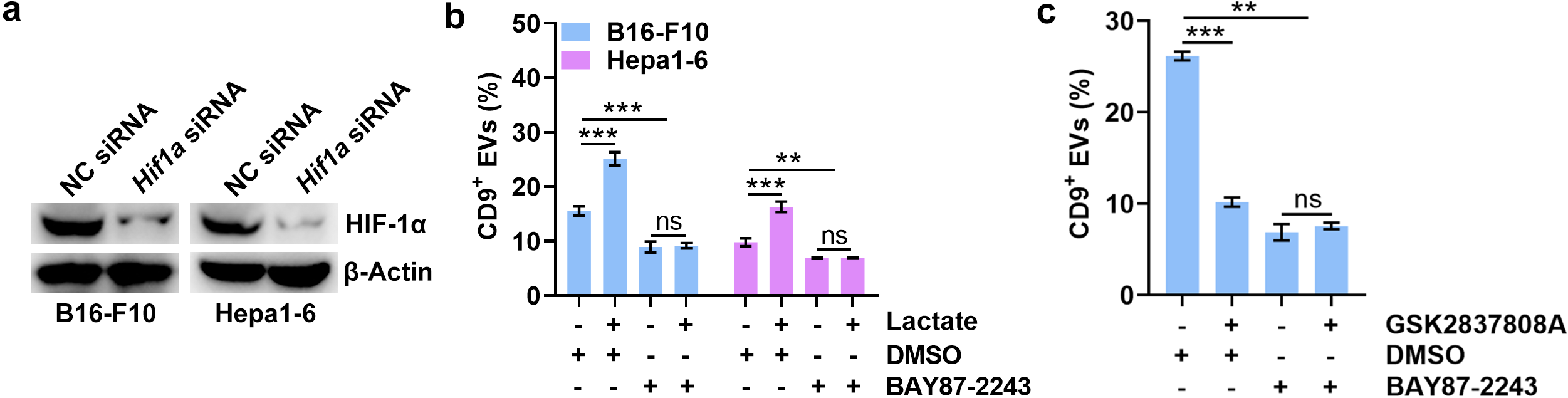


**Figure S2.** Lactate promotes sEV release in a HIF-1α-dependent manner. (a, b) After transfection of *Hif1a-*specific siRNA for 24 h, the HIF-1α protein level in B16-F10 cells was detected by western blotting (a). B16-F10 EVs and Hepa1-6 EVs were isolated from the supernatants of these cells, and then the excessive sEVs were adsorbed onto latex beads, followed by CD63 detection on these sEVs by flow cytometry. (c-f) In the presence of 100 nM BAY87-2243, B16-F10 and Hepa1-6 cells were treated with or without 10 mM lactate for 24 h (c, d), or B16-F10 cells were treated with 1 μM GSK2837808A for 24 h (e, f). Then, the sEVs in the culture supernatant were captured with anti-CD63-coated latex beads, and the percentage of CD9^+^ sEVs was determined by flow cytometry and statistically analyzed (c, e). The sEVs in the culture supernatant were isolated, and the particle concentration was measured by NTA (d, f). Data are shown as the mean ± SD of one representative experiment (n = 3). Similar results were observed in three independent experiments. One-way ANOVA followed by Tukey’s test. **P* < 0.05; ***P* < 0.01; ****P* < 0.001; ns, not significant.


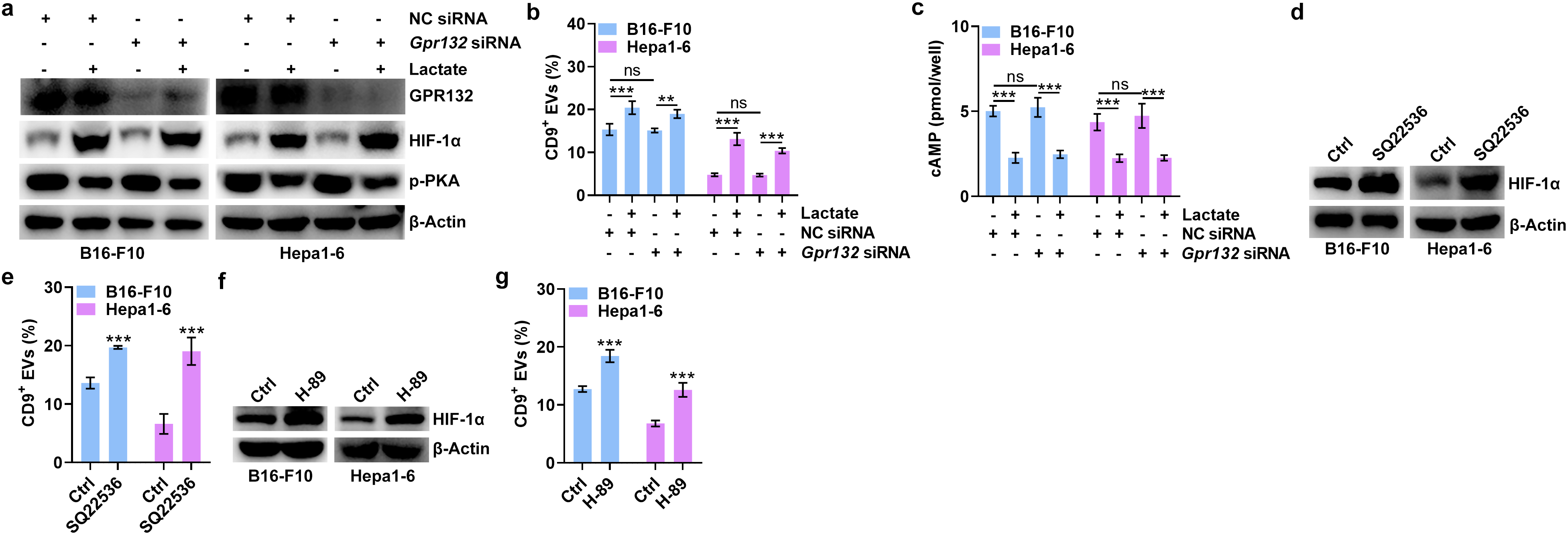


**Figure S3.** Lactate upregulates HIF-1α via GPR81-mediated inhibition of the cAMP/PKA axis. (a-c) After transfection with *Gpr132*-specific siRNA for 24 h, B16-F10 and Hepa1-6 cells were treated with or without 10 mM lactate for another 24 h. Then, the protein levels of GPR132, HIF-1α, p-PKA and β-Actin in both cell lines were detected by western blotting (a). The sEVs in the culture supernatant were captured with anti-CD63-coated latex beads, and the percentage of CD9^+^ sEVs was determined by flow cytometry and statistically analyzed (b). The intracellular cAMP concentration was measured (c). (d, e) B16-F10 and Hepa1-6 cells were treated with or without 1 mM SQ22536 for 24 h. Then, HIF-1α and β-Actin in both cell lines were detected by western blotting (d). The sEVs in the culture supernatant were captured with anti-CD63-coated latex beads, and the percentage of CD9^+^ sEVs was determined by flow cytometry and statistically analyzed (e). (f, g) B16-F10 and Hepa1-6 cells were treated with or without 10 μM H-89 for 24 h. Then, HIF-1α and β-Actin in both cell lines were detected by western blotting (f). The sEVs in the culture supernatant were captured with anti-CD63-coated latex beads, and the percentage of CD9^+^ sEVs was determined by flow cytometry and statistically analyzed (g). Data are shown as the mean ± SD of one representative experiment (n = 3). Similar results were observed in three independent experiments. One-way ANOVA followed by Tukey’s test in (b, c); unpaired Student’s *t* tests in (e, g). ***P* < 0.01; ****P* < 0.001; ns, not significant.


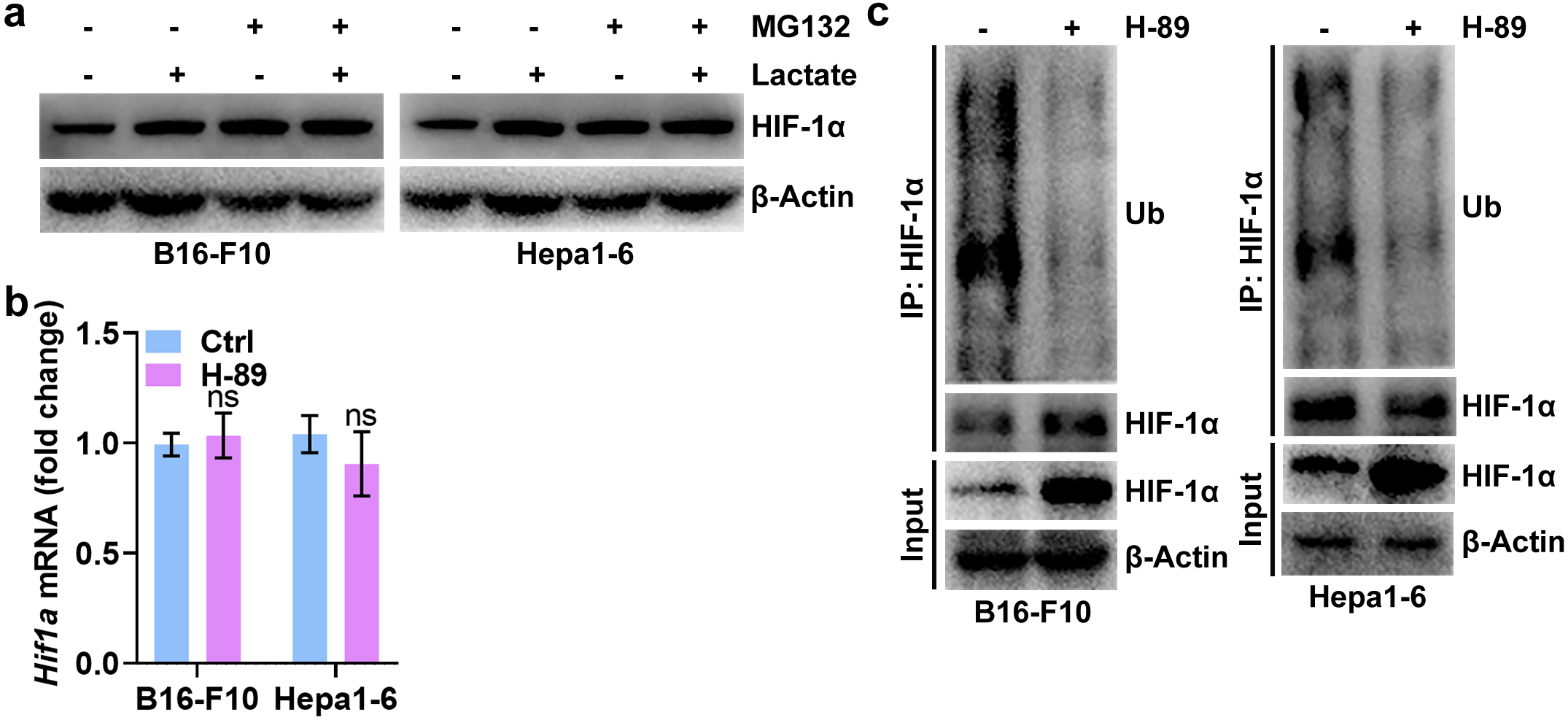


**Figure S4.** Lactate inhibits the PKA-mediated ubiquitination HIF-1α that causes degradation. (a) In the presence of 20 μM MG132, B16-F10 and Hepa1-6 cells were treated with or without 10 mM lactate for 12 h, and then the HIF-1α protein level was detected by western blotting. (b, c) B16-F10 and Hepa1-6 cells were treated with or without 10 μM H-89 for 24 h, and the *Hif1a* mRNA level in both cell lines was measured by real-time PCR. The *Hif1α* expression levels in Ctrl groups were set as 1, which was used as a baseline for other groups (b), or the K48-linked polyubiquitination of HIF-1α in both cell lines was detected by western blotting (c). Data are shown as the mean ± SD of one representative experiment (n = 3). Similar results were observed in three independent experiments. Unpaired Student’s *t* tests. ns, not significant.


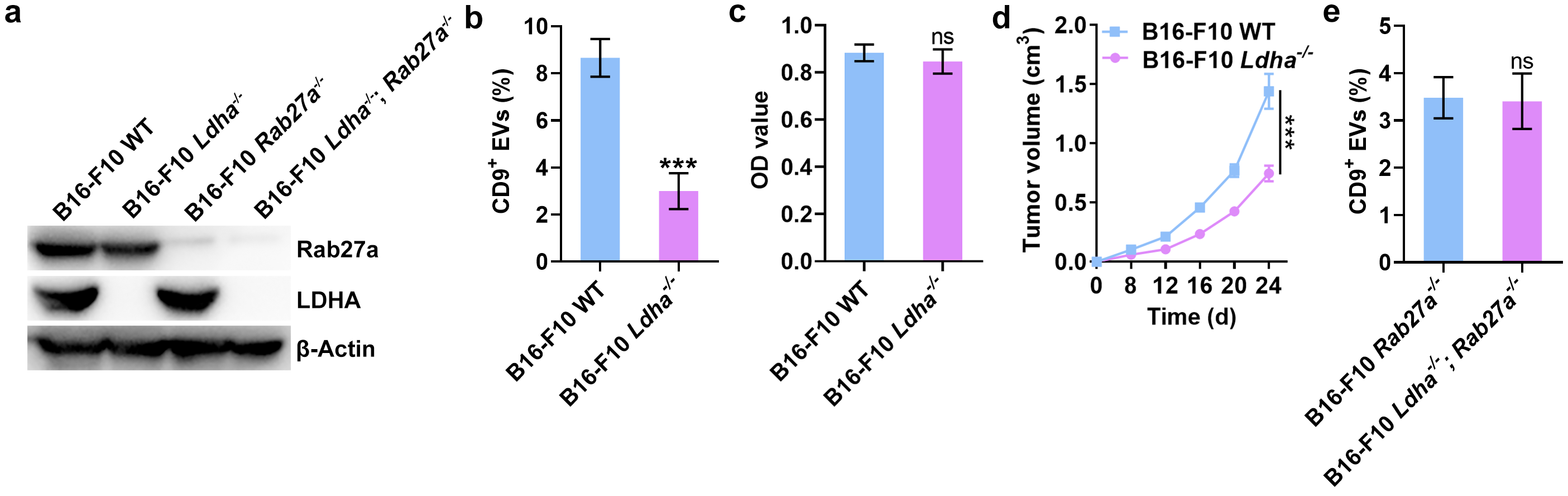


**Figure S5.** Lactate promotes tumor progression by inducing sEV release from tumor cells. (a) The protein levels of LDHA and Rab27a in B16-F10 WT, B16-F10 *Ldha^-/-^*, B16-F10 *Rab27a^-/-^* and B16-F10 *Ldha^-/-^*; *Rab27a^-/-^* cells were detected by western blotting. (b, c) B16-F10 WT and B16-F10 *Ldha^-/-^* cells were cultured for 24 h. Then, the sEVs in the culture supernatants of these cells were captured with anti-CD63-coated latex beads, and the percentage of CD9^+^ sEVs was determined by flow cytometry and statistically analyzed (b). Cell proliferation was measured with a CCK-8 assay (c). (d) Mice were subcutaneously injected with B16-F10 WT or B16-F10 *Ldha^-/-^* cells on Day 0. Then, tumor size was measured and statistically analyzed on Day 24. (e) B16-F10 *Rab27a^-/-^* and B16-F10 *Ldha^-/-^*; *Rab27a^-/-^* cells were cultured for 24 h. Then, the sEVs in the culture supernatants of these cells were captured with anti-CD63-coated latex beads, and the percentage of CD9^+^ sEVs was determined by flow cytometry and statistically analyzed. Data are shown as the mean ± SD of one representative experiment (n = 5). Similar results were observed in three independent experiments. Unpaired Student’s *t* tests. ****P* < 0.001; ns, not significant.


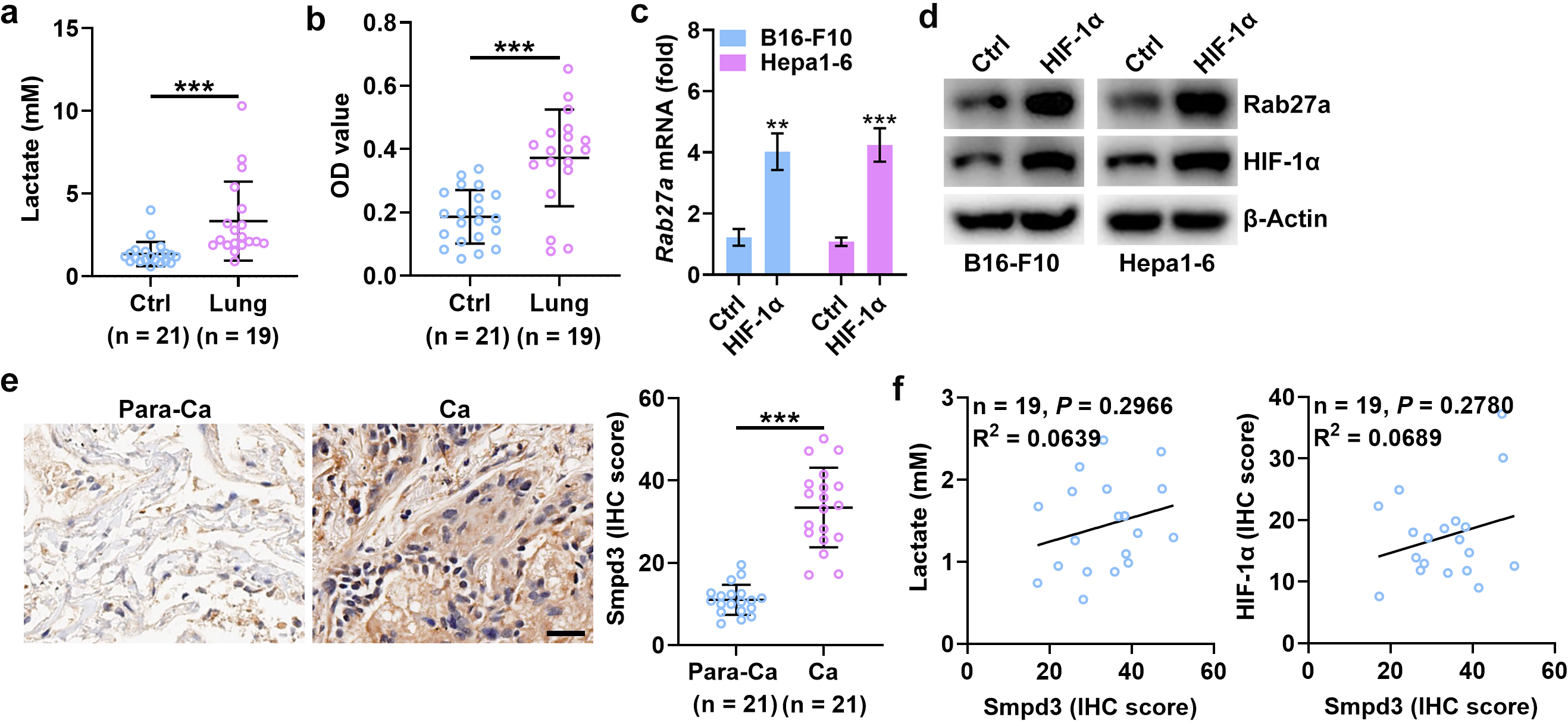


**Figure S6.** Plasma lactate and sEVs of cancer patients are positively correlated. (a, b) The levels of lactate (a) and sEVs (b) in the plasma of healthy controls and lung cancer patients were statistically analyzed. (c, d) B16-F10 and Hepa1-6 cells were overexpressed with HIF-1α for 24 h (c) or 48 h (d). Rab27a mRNA (c) and protein (d) levels in these cells were detected by RT-PCR (c) and western blotting (d). (e) Smpd3 protein levels in Ca and Para-Ca of lung cancer patients were detected by IHC staining (left) and statistically analyzed. Scale bar, 20 μm. (f) Correlation analysis of the indicated factors. Data are shown as the mean ± SD. Unpaired Student’s *t* tests in (a-c, e); Spearman correlation analysis in (f). ****P* < 0.001; ns, not significant.

| Supplementary Table 1: Clinical characteristics of lung cancer patients (n = 19) | |
| --- | --- |
| Characteristics | **Number of patients** |
| Gender |  |
| Male | 13 |
| Female | 6 |
| Age |  |
| ≤ 50 | 4 |
| 50-75 | 13 |
| > 75 | 2 |
| Histological subtype |  |
| Small cell lung cancer | 2 |
| Non-small cell lung cancer | 17 |
